# Automated Continuous Crystallization Platform with Real-Time Particle Size Analysis via Laser Diffraction

**DOI:** 10.1021/acs.oprd.4c00110

**Published:** 2024-07-09

**Authors:** Sayan Pal, Arun Pankajakshan, Maximilian O. Besenhard, Nicholas Snead, Juan Almeida, Shorooq Abukhamees, Duncan Craig, Federico Galvanin, Asterios Gavriilidis, Luca Mazzei

**Affiliations:** † Department of Chemical Engineering, University College London, Torrington Place, London WC1E 7JE, U.K.; ‡ Perceptive Engineering, Applied Materials, Vanguard House, Keckwick Lane, Sci Tech Daresbury, Cheshire WA4 4AB, U.K.; § Department of Pharmaceutics and Pharmaceutical Technology, Faculty of Pharmaceutical Sciences, The Hashemite University, Zarqa 13115, Jordan; ∥ Faculty of Science, University of Bath, Claverton Down, Bath BA2 7AY, U.K.

**Keywords:** antisolvent crystallization, automation, ibuprofen, process analytical technology, automated laser diffraction, online, LabVIEW, PharmaMV

## Abstract

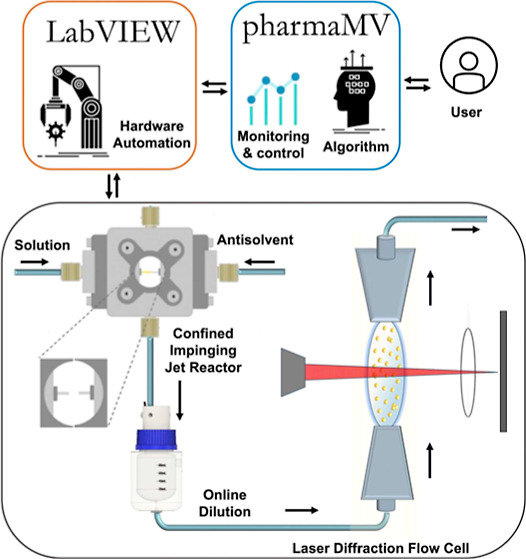

The fourth industrial revolution is gaining momentum in the pharmaceutical industry. However, particulate processes and suspension handling remain big challenges for automation and the implementation of real-time particle size analysis. Moreover, the development of antisolvent crystallization processes is often limited by the associated time-intensive experimental screenings. This work demonstrates a fully automated modular crystallization platform that overcomes these bottlenecks. The system combines automated crystallization, sample preparation, and immediate crystal size analysis via online laser diffraction (LD) and provides a technology for rapidly screening crystallization process parameters and crystallizer design spaces with minimal experimental effort. During the LD measurements, to avoid multiple scattering events, crystal suspension samples are diluted automatically. Multiple software tools, i.e., LabVIEW, Python, and PharmaMV, and logic algorithms are integrated in the platform to facilitate automated control of all the sensors and equipment, enabling fully automated operation. A customized graphical user interface is provided to operate the crystallization platform automatically and to visualize the measured crystal size and the crystal size distribution of the suspension. Antisolvent crystallization of ibuprofen, with ethanol as solvent and water with Soluplus (an additive) as antisolvent, is used as a case study. The platform is demonstrated for antisolvent crystallization of small ibuprofen crystals in a confined impinging jet crystallizer, performing automated preplanned user-defined experiments with online LD analysis.

## 1 Introduction

Antisolvent crystallization is a widely used and efficient process for the manufacturing of small crystal suspensions. Crystallization of the solute is accomplished by adding an antisolvent that reduces the solubility of the solute. The method is used for a wide range of materials, such as active pharmaceutical ingredients (APIs), inorganic and organic crystals, polymers, and proteins.^1−3^ The prevailing batch synthesis techniques of API suspensions by means of antisolvent precipitation make process development a time, manpower, and raw material intensive task, with challenges for scalable production. This sparked significant interest in the development of continuous crystallization technologies, which has grown over the last two decades. A continuous antisolvent crystallization system results in consistent and better product quality, including crystal size and morphology, than batch counterparts,^4−7^ with the production of API crystals with consistent properties throughout the manufacturing process being a main driver for continuous crystallization.^8^

Although continuous stirred tank reactors (CSTRs) in series,^9,10^ CSTRs operated in mixed-suspension mixed-product removal mode,^8,11^ plug flow crystallizers,^12^ and oscillatory baffled crystallizers^13^ have been reported to be robust antisolvent crystallizers, recently impinging jet reactors^4,14^ have gained popularity, providing process intensification pathways to systems involving rapid precipitation, especially when high supersaturation is needed and mixing time must be faster than induction time. In impinging jet reactors, at least two liquid jet streams collide at high velocities, their kinetic energy converting into chaotic motion via impingement and redirection of the flow over a tiny volume.^15^ Impinging jet reactors have been reported to provide excellent mixing (with mixing times as low as few milliseconds), and over the last two decades, they have been used as robust continuous antisolvent crystallizers.^4,15,16^ Despite its advantages, the application of impinging jet reactors in continuous crystallization has been limited due to the short residence times of these reactors and the fact that they are suitable only for systems with fast crystallization kinetics.^14^

Paradoxically, even though the development of continuous flow systems for antisolvent crystallization has thrived, crystallization processes are still developed and optimized predominantly in batch reactor systems. One of the reasons for this is related to the complexity of characterizing particle properties with process analytical technology (PAT). Although over the past few years spectroscopy- and chromatography-based PAT have been reported for particulate processes such as crystallization,^17−19^ its widespread application is limited owing to requirements of specific reactors and analytical techniques that require sample preparation.

Continuous process development still remains a roadblock owing to the lack of PAT for crystal size characterization. The complex and multivariable nature of antisolvent crystallization and its optimization make the search and discovery of optimal operating conditions challenging. Antisolvent crystallization of APIs can be tuned by manipulating various input parameters,^4^ but identifying suitable conditions for targeting a specific size of API crystals in the multivariable design space is highly complex. To reduce the experimental effort and navigate the design space efficiently with the powerful tools of design of experiments (DoE),^20,21^ PAT for crystal size characterization is essential. In laboratory experimentation, automating reactor systems, particularly antisolvent crystallization platforms, has the potential to save considerable time and effort. Automated antisolvent crystallization platforms can be used to conduct preplanned experiments for rapid screening of experimental conditions, additives, and different reactors. Automation of a crystallization platform is also a prerequisite for developing autonomous (that is, self-optimizing and closed-loop) systems with predictive modeling based on machine learning algorithms and digital decision-making methods, which can dramatically improve the way parameter spaces are explored.

Most particle characterization technologies for particle suspensions, e.g., dynamic light scattering, laser diffraction (LD), electron and optical microscopy, and nanoparticle tracking analysis, are mainly implemented offline because they require sample preparation and dilution. Laser-based methods, such as focused beam reflectance measurement (FBRM) probes, are renowned^22^ for inline crystal size characterization in terms of chord length distributions. In addition to FBRM probes, the Mettler Toledo’s particle video microscope probe and the BlazeMetrics’ Blaze 400, both of which provide in situ digital micrographs, are adopted as a direct visual method for measuring particle sizes.^23^ However, such techniques are typically limited to large particle sizes, can be misleading in terms of the actual particle sizes,^23^ and are limited to the field of view of the probe. In addition, FBRM requires complex modeling steps^24^ to calculate particle sizes from chord lengths and is valid only for well-defined particle shapes. Moreover, such methods are usually only applicable in batch/semibatch reactors or CSTRs due to their probe-based designs, making them unsuitable for other systems, such as plug flow crystallizers. Neugebauer et al.^25^ employed a microscope equipped with a flow cell, along with image analysis algorithms for particle tracking and shape analysis, for a crystallization process carried out in segmented flow. Kacker et al.^26^ demonstrated a high-resolution monocular microscope probe developed by SOPAT GmbH for inline measurement of particle sizes at the exit of a continuous oscillatory flow baffled crystallizer. Despite the recent developments in imaging-based process analytical technologies for particle size distributions (PSDs), these methods do require complex image processing algorithms and do not provide a volume-based PSD (the type of distribution usually favored in pharmaceutical applications), which instead can be obtained from the well-established LD technique. LD benefits from the broad particle size range (from submicrometers to millimeters) and provides better statistics than most optical methods. The wide size range is especially useful at small (<10 μm) particle sizes, where the aforementioned optical methods typically fail. Latest developments from Malvern,^27^ Beckman Coulter,^28^ and Sympatec^29^ based on multiple laser systems extend the particle size limits down to 10 nm.

Even if LD has been commonly used as offline characterization technique, its implementation as PAT has been uncommon^30,31^ and mainly used for dry powders.^32^ Handling of crystal suspensions, especially in an automated fashion, is the principal challenge in developing an LD system as PAT because such system requires suitable pumps, valves, dilution systems, and flow cells. Typically, crystal suspensions must be suitably diluted to avoid errors from multiple light scattering,^33^ as LD uses the angular dependence of the light scattered by the particles to estimate the PSD.^26^ This is another key challenge when LD is implemented online. Hence, a standard LD system has not yet been developed to be implemented as PAT, despite the absence of limitations for automated analysis inherent to the measurement principle itself. This work overcomes these obstacles by using suitable pumps and valves to automatically collect and dilute the samples that elute from a flow crystallizer in a collection vessel prior to flowing to the optical flow cell of the LD analyzer.

This work demonstrates the production of API crystal suspensions via continuous antisolvent crystallization with integrated online particle characterization via LD. The platform is fully automated and used to produce ibuprofen crystal suspensions in a confined impinging jet reactor (CIJR). This system was found to be robust, provides excellent mixing, and results in consistent product quality (crystal size). The graphical user interface (GUI) developed for the crystallization platform can control all the hardware in the platform, from pumps to valves, and the LD system, all integrated in one software framework that allows easy operation and automatic runs of preplanned user-defined experiments. The online LD measurement system was validated using particle standards and subsequently demonstrated for continuous crystallization of ibuprofen.

A final clarification is in order. Full automation means that once a list of experiments is given to the system and a start button is clicked, the experimental setup can perform the experiments and visualize the results without any user intervention. In contrast, fully autonomous means that the user only provides the ranges within which the input variables of the experimental platform should vary, and upon clicking the start button, the platform identifies which initial experiments to run; it then analyzes the experimental data and employs machine learning to develop a data-driven model to decide which experiments to perform next to better screen the design space and refine the model representing the system, all without any user intervention. This work focuses on the development of a fully automated crystallization platform only.

## 2 Materials and Methods

### 2.1 Crystallization Platform

#### 2.1.1 CIJR Used as a Crystallizer

**Figure 1:**
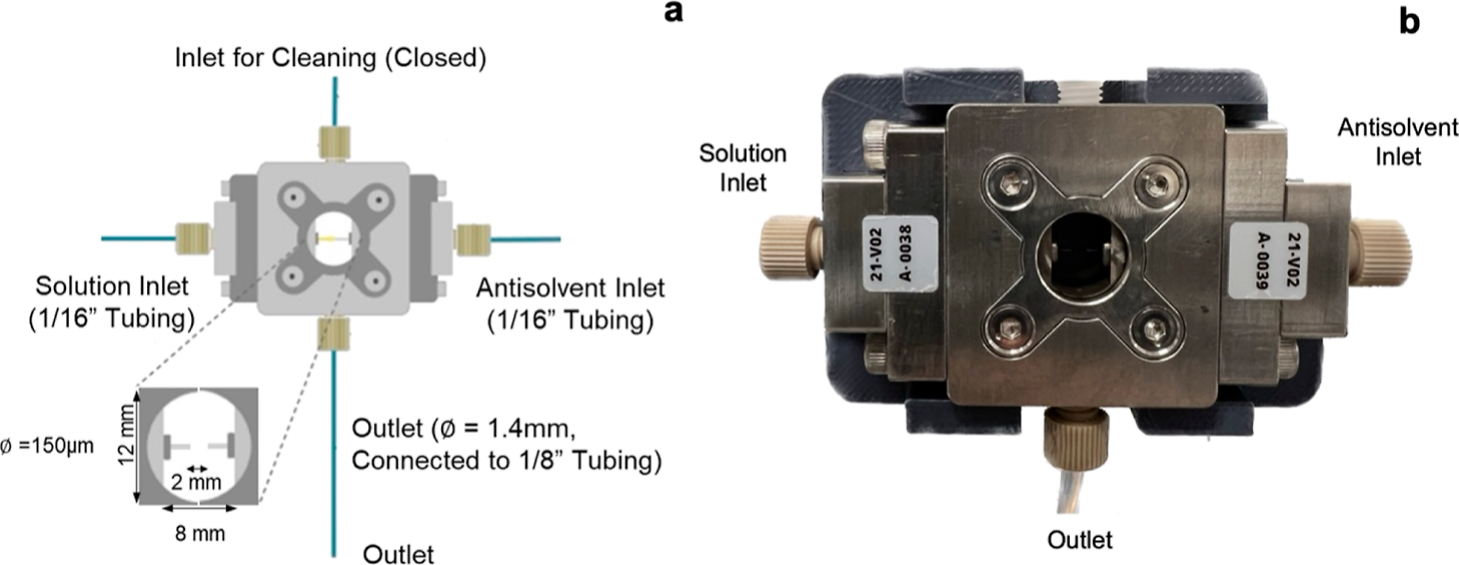
CIJR. (a) Schematic and (b) image.

The CIJR used in the current work was fabricated by Fluigent (France) by modifying their commercially available RayDrop droplet generator. The CIJR consists of three main removable parts: two equal impinging jet capillary inserts (150 μm diameter) on each side, a metal reactor enclosure with glass windows, and an outlet capillary at the bottom. There were four standard microfluidic connections (1/4″-28): two on the jet capillary inserts for the solution and antisolvent inlets, one on the crystal suspension collecting outlet, and a closed top connection for cleaning (Figure 1). The CIJR was attached to the crystallization platform rig via a 3D printed holder, which allowed for visual inspection during the experiments.

#### 2.1.2 Downstream Sampling and Online LD

**Figure 2:**
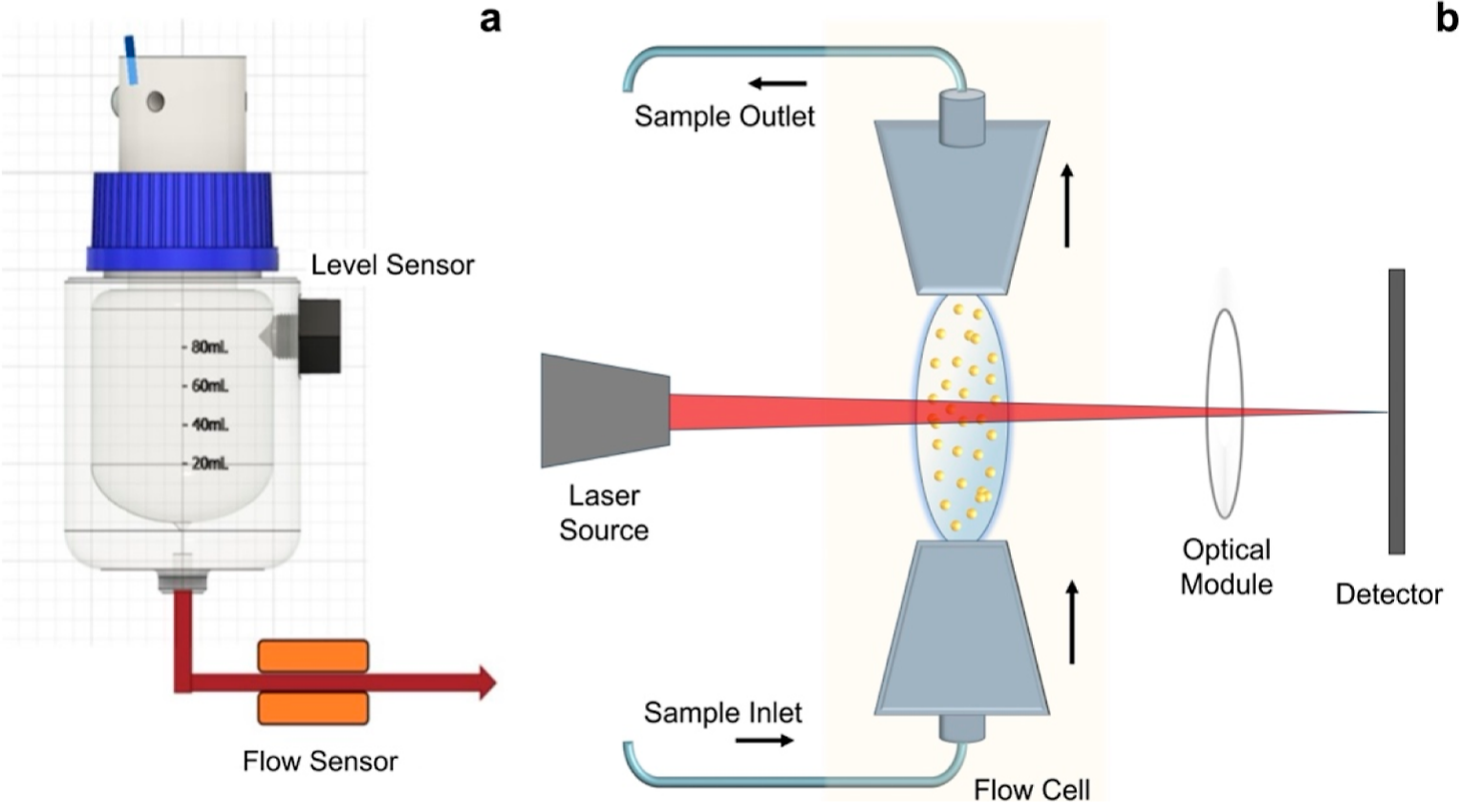
Schematic of (a) sample vessel 2 (SV2) and (b) LD flow cell.

Two sample vessels were used in the experimental setup, SV1 and SV2, for sample collection from the CIJR and for sample dilution for LD measurements, respectively. The vessels were designed with a concave bottom surface and bottom outlet (Figure 2a) and fabricated by 3D printing (Formlabs Form 3+) using a Formlabs clear resin material. The top of the vessels was designed with a GL45 thread, which fits commercially available solvent bottle caps with check valves. All inlet and outlet ports of vessels SV1 and SV2 had 1/4″-28 flat bottom port configurations to match standard commercially available fluidic connectors. A 3D printed stage was fabricated for mounting the sample vessels at the desired height on top of magnetic stirrers (IKA Digital), ensuring proper stirring.

Two fiber optic-based level sensors (Keyence FS N-40) were threaded into the two 3D printed vessels to detect the liquid level. Flow sensors (Sensirion SLF3x) were used at the outlets of SV2 and the LD flow cell to sense when these were empty during the LD crystal size analysis process. A pneumatically actuated four-way Swagelok ball valve (Swagelok SPL43Y) was used for feeding samples from SV1 into SV2. A USB controlled eight-channel mechanical relay was used for sending the power signal to the pneumatic actuator to switch the valve.

An LD analyzer (Beckman Coulter, LS320) with a customized flow cell was used for LD measurements (Figure 2b). The inlet and outlet connections to the LD flow cell were modified to fit standard Swagelok fittings (1/8′). To connect the flow cell to the sample vessel SV2, the standard PTFE tubings (10 mm diameter) were replaced with 1.58 mm ID PTFE tubing (VICI Jour); this resulted in a significant decrease in the volume of diluted sample needed for each LD measurement cycle, making the measurements faster. The required amount of sample dilution employed for the measurements was determined via obscuration—a parameter measured by the LD analyzer. The concentration of particles for which the obscuration is in the range of 5 to 20% is optimal for measurement reliability. The amount of dilution required to achieve an obscuration inside the optimal range for LD measurements was obtained from initial manually performed experiments and kept constant for experiments with the same antisolvent/solvent ratio. To measure particle size reliably, we ensured that all the experiments were performed in the recommended obscuration range (5–20%). For every LD measurement of particle size, the obscuration was also measured and saved in a.csv file. During post-processing of the data, if a measurement was found where the obscuration value was not in the recommended range, that experiment was repeated with higher or lower dilution as required. For the LD measurements, the Mie theory optical model was used, with 0.01 as the imaginary part of the sample refractive index.

#### 2.1.3 Materials

For the crystallization of ibuprofen, ethanol was used as the solvent and deionized (DI) water as the antisolvent. The ibuprofen solution (1 wt % of equilibrium solubility) was obtained by dissolving 3.95 g of ibuprofen in 100 mL of ethanol. Soluplus (BASF), a polymeric solubilizer, was added to the antisolvent as an additive for crystal growth inhibition and stabilization. The stock additive solution was prepared by dissolving 5 g of Soluplus in 100 mL of DI water using ultrasonication followed by magnetic stirring. For background measurement in the LD analyzer, a mixture of antisolvent and solvent was used, mixed in the same ratio as the antisolvent/solvent ratio employed for the crystallization experiments. To dilute the samples, a saturated ibuprofen solution was used; it was prepared by adding 10 mg of ibuprofen to 500 mL of the background solution by stirring overnight and by filtering the undissolved particles using 0.22 μm PVDF syringe filters. For the initial validation of the developed online LD analysis system, 2 and 15 μm sized monodisperse polystyrene particle standards (Sigma-Aldrich) were used.

### 2.2 Online Sampling Process for LD

**Figure 3:**
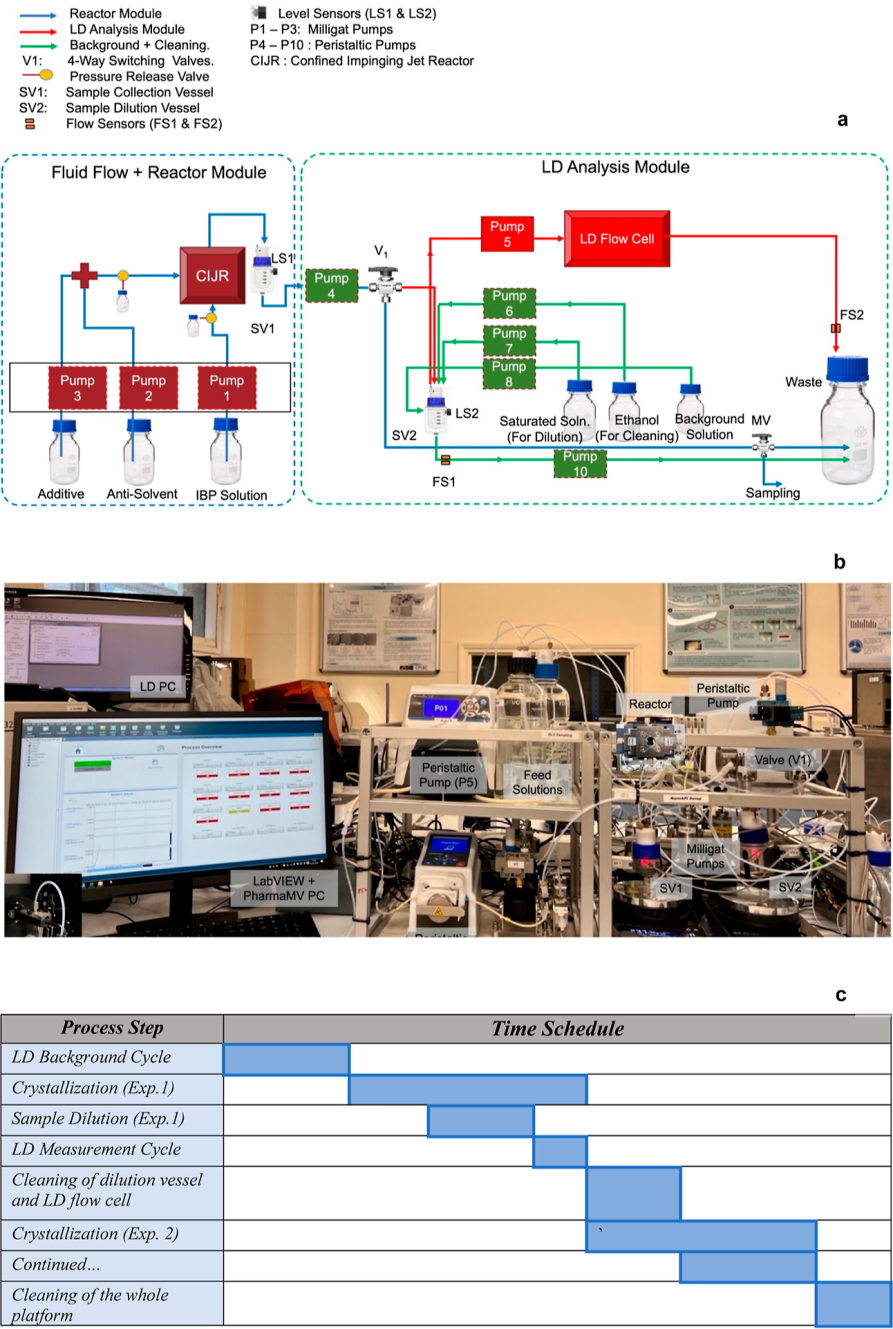
(a) Process flow diagram, (b) picture, and (c) sequence of steps during the routine operation of the crystallization platform with online LD analysis.

The process flow diagram and an image of the automated continuous crystallization platform with online LD particle size analysis are shown in Figure 3a,b, respectively. Operation involved first running a background cycle for the LD instrument and then performing a list of preset antisolvent crystallization experiments. This also included automated cleaning of the online LD analysis section (shown in green on the process flow diagram) and semiautomated cleaning of the reactor section (shown in blue on the process flow diagram). Figure 3c shows the steps performed sequentially during automated crystallization experiments followed by online LD analysis, and all of the pieces of equipment with their respective functions and online control strategy are presented in Table 1. As shown in Figure 3a, pumps 1, 2, and 3 were used for feeding the ibuprofen solution, the antisolvent, and the additive solution to the CIJR, respectively. Two pressure release valves (IDEX Health Sciences, 100 PSI) were used on the two inlets of the CIJR to prevent any damage to the reactor in the case of reactor fouling. The crystallized suspension from the CIJR outlet was collected in SV1. When SV1 was filled (detected by level sensor LS1), pump 4 was instructed to flow the suspension from SV1 to a waste/sampling bottle, operating at a flow rate equal to the total flow rate of pumps 1–3 and maintaining a constant liquid level in SV1. Then, pump 7 was instructed to fill SV2 with saturated ibuprofen solution until it was detected to be full by level sensor 2 (LS2). Valve V1 was instructed to obtain suspension samples from the process stream periodically. This stream was diluted and stirred in SV2 using a magnetic stirrer to homogenize the mixture. The amount of dilution required for LD measurements can be controlled by varying the volume of sample pumped from SV1 to SV2, and the valve V1 switching time was adjusted accordingly. The volume of saturated solution added to the sample dilution vessel (SV2) was kept constant, and the volume of sample added by switching valve V1 was increased or decreased to control the dilution. Subsequently, the diluted sample was pumped into the LD flow cell by pump 5. When the flow cell was detected to be filled up by the diluted solution (using FS2), the LD measurement was initiated. After the LD measurement of the diluted sample was completed and the flow cell and SV2 were emptied (detected by FS2), pump 5 was stopped, and any remaining solution in SV2 was emptied from the bottom of the vessel using pump 10. A cleaning liquid (ethanol–water solution) was utilized to fill SV2 using pump 6 and flowed through the LD flow cell using pump 5. After the cleaning cycle was completed, the next LD measurement cycle was initiated for the next set of preplanned experimental parameters. Pump 8 was used to feed the background solution to SV2 before the LD measurement process. Standard PEEK connectors (IDEX Health Science) and Teflon tubings (VICI Jour) were used to connect the reservoirs, pumps, valves, and sample vessels.

**Table 1:** Devices in the Crystallization Platform, along with Their Function, Data Communication Software, and Manufacturer

nomenclature	device name	functions	communication	manufacturer
P1, P2, P3	milliGAT pump	feed solutions to crystallizer	LabVIEW drivers	GlobalFIA
P4, P6–P10	Ismatec Reglo 4-channel peristaltic pump	transfer samples and cleaning solution	LabVIEW drivers	Masterflex
P5	Masterflex peristaltic pump	feed sample to and clean LD flow cell	LabVIEW drivers	Masterflex
V1	valve	switch flow path	Numato USB relay with LabVIEW driver	Swagelok
LS1, LS2	level sensor	detect liquid level in sample collection and dilution vessels	Arduino-based analogue to digital converter device	Keyence Inc.
FS1, FS2	flow sensor	detect flow	LabVIEW drivers	Sensirion
MS1, MS2	magnetic stirrer	stir sample collection and dilution vessels	LabVIEW drivers	IKA

A manual Swagelok valve (MV), placed on the blue line going from valve V1 to the waste collection bottle, was used to collect samples for validation and other analysis. After the screening of the operating parameters was completed, the continuous crystallization platform was operated entirely for production without involving the analysis section of the process flow diagram (see Figure S4 in the Supporting Information). A separate collection bottle was added at the end of blue line, keeping valve V1 in that direction. The setup can be very easily modified based on the goal—screening or production.

Although most of the devices in the crystallization platform had a digital interface, an analogue to a digital converter system was developed for analogue devices, such as the pneumatically actuated four-way valve (V1) and the two level sensors (LS1 and LS2). A USB relay was employed that sent the desired voltage to open and close the valve V1. The level sensors were calibrated to send a 4 V signal when the liquid in the sample vessels reached the desired level. An Arduino-powered DAC system was used to detect the analogue voltage signal from the fiber optic level sensor and convert it to a digital binary signal.

## 3 Automation of the Crystallization Platform

The three main components involved in the automation of the crystallization platform with online LD are (i) instrument interfacing, which facilitates instrument control from a computer, (ii) data communication protocol, for secure information exchange within and across the platform, and (iii) process automation, which involves implementation of an event flow and interactive visualization environment. In this section, these components are explained in detail.

### 3.1 Instrument Interfacing

**Figure 4:**
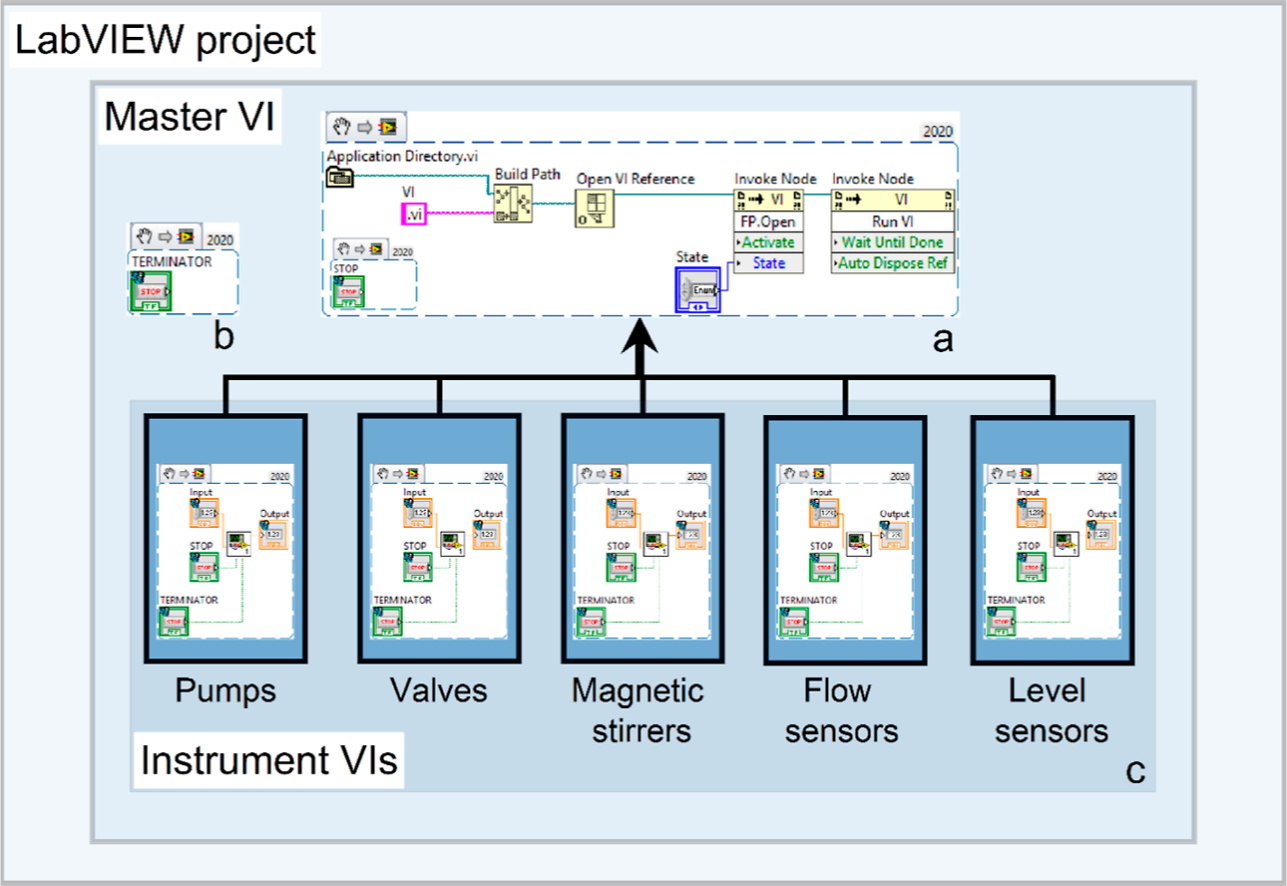
Illustration of the LabVIEW program development for interfacing all of the hardware components of the crystallization platform.

All of the instruments of the crystallization platform described in Table 1 were connected to a PC through data acquisition devices (DAQs) such as serial communication, USB, and Ethernet. The DAQs and the instrument drivers were used to develop graphical codes, called virtual instruments (VIs) in LabVIEW,^34^ that can be then used to configure, program, and troubleshoot the instruments. The LabVIEW code architecture developed for automating instrument functions is illustrated in Figure 4. The code includes a LabVIEW project that houses individual VIs for pumps, valves, magnetic stirrers, flow sensors, and level sensors. Additionally, a master VI was created to control all of these VIs collectively. A snippet of the LabVIEW code used in the master VI to manage the instrument VIs is labeled as (a) in Figure 4, and further details on VI development are provided in the Supporting Information (Section S1).

For the LD analyzer, a LabVIEW-based protocol was unavailable for online control of the equipment; therefore, an Open Platform Communications Unified Architecture (OPC UA) server was developed in Python to start the background and measurement cycles in the LD software by performing automated mouse clicks.

### 3.2 Data Communication Protocol

**Figure 5:**
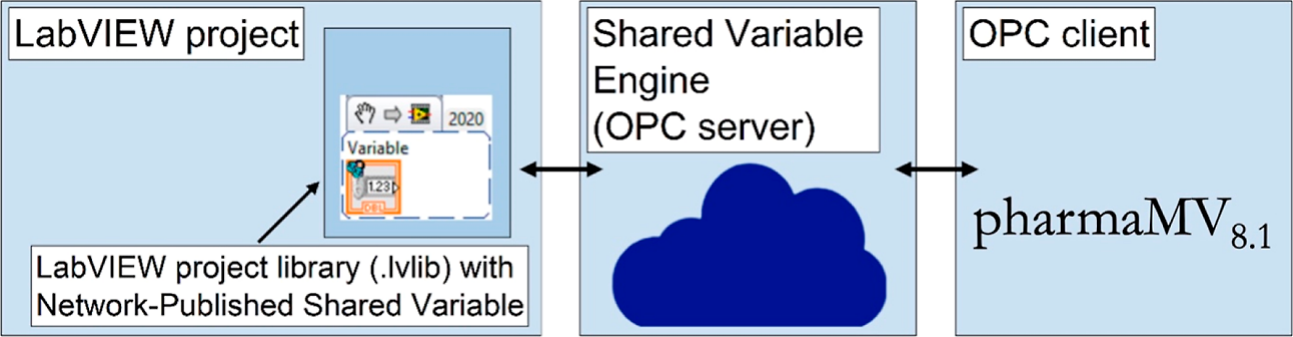
Schematic of the secure data communication protocol for the automated crystallization platform based on OPC DA.

**Figure 6:**
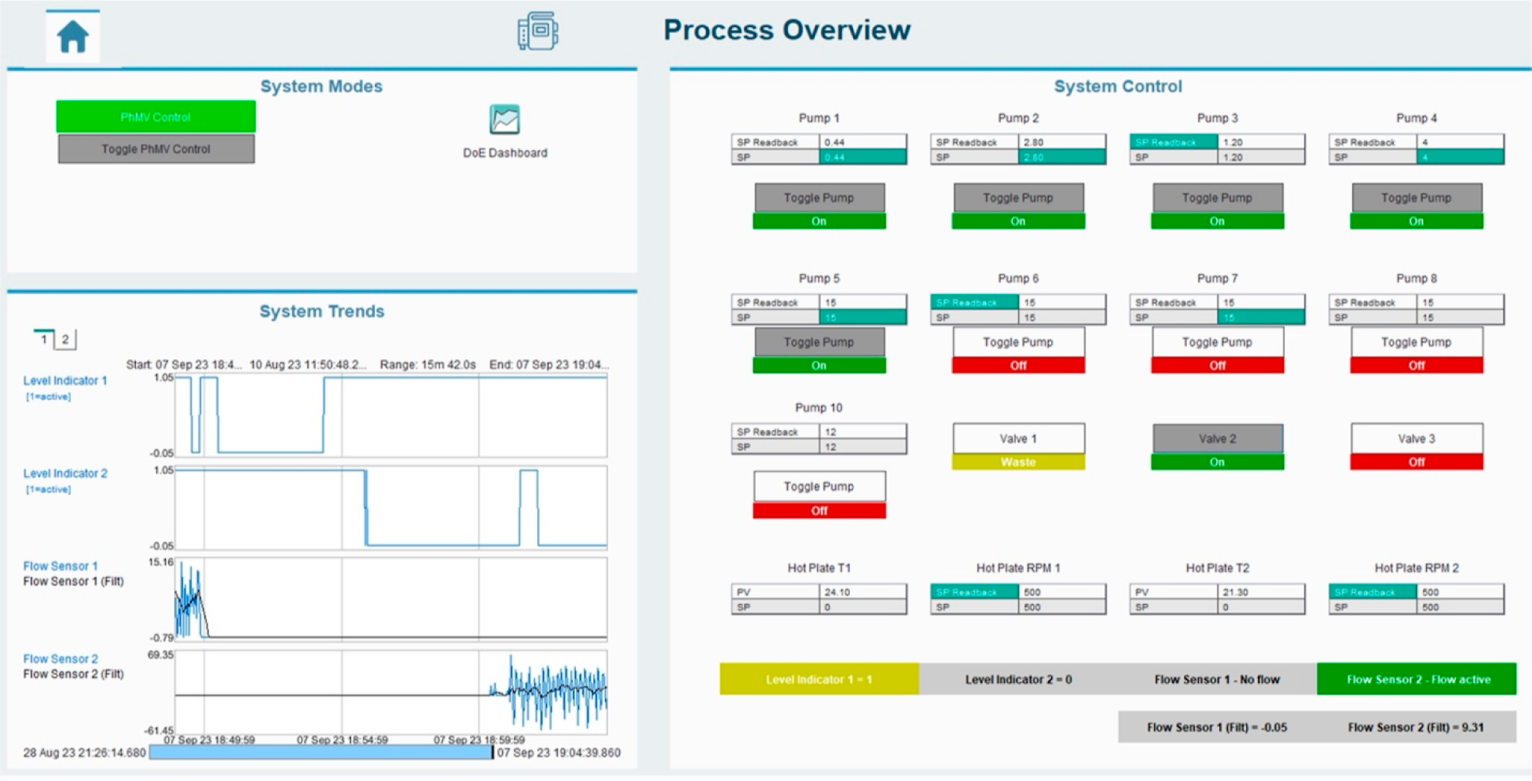
GUI in PharmaMV for controlling and monitoring the entire crystallization platform.

The input–output data of the instrument VIs were defined as network-published shared variables, which can send data over a network through a software component called the shared variable engine (SVE). On deployment, SVE acts as an OPC server,^35^ creating OPC tags for all the process variables and making them securely accessible to PharmaMV,^36^ a software developed by Perceptive Engineering as open platform communication data access (OPC DA) client. Figure 5 summarizes the data communication protocol used; further details on the data communication protocol are given in Section S2 of the Supporting Information. In the current work, PharmaMV was used for process automation and development of the GUI for controlling the crystallization platform. The GUI, which can be used to control all the devices in the crystallization platform, is shown in Figure 6.

### 3.3 Process Automation

**Figure 7:**
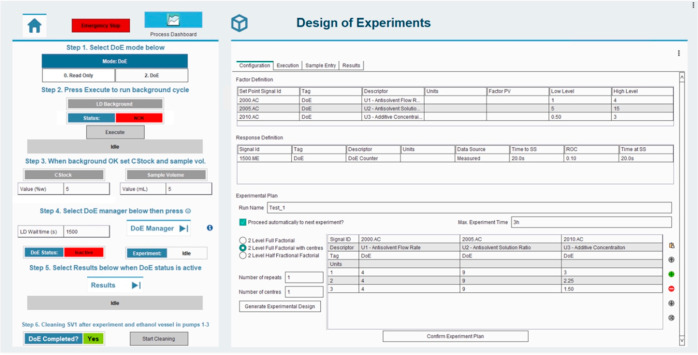
GUI in PharmaMV for performing the preplanned fully automated crystallization experiments with online automated LD measurements.

Individual Python scripts were developed and employed in PharmaMV for performing preplanned experiments, background measurement cycles for LD, analysis cycles for LD, and cleaning cycles using timed loop and sensor readings. The duration of each experimental run, which, in addition to the time necessary for the steady-state synthesis of the crystals, included the times required for sample preparation in SV2, online LD analysis, and cleaning of the LD flow cell and SV2, was set at 25 min. The GUI for performing the various steps of the process flow diagram (reported in Figure 3c) in a fully automatic fashion was developed in PharmaMV and is shown in Figure 7. The system trends displayed on the GUI are the signals read from the flow and level sensors, and the system controls displayed on the GUI are those for different pieces of hardware in the crystallization platform. The DoEs block in the GUI (see Figure 7) was employed to set the list of preplanned experimental conditions, and the PharmaMV scripts were developed to calculate the feed pump flow rates required to perform the respective experiments. In the automated procedure, the LD analyzer automatically saved the measured data in a.csv file for each experiment in a network shared folder. A PharmaMV script was used to access these files, to read and display the results in the GUI, and to change the experimental conditions to the next preplanned experiment, upon detecting a new.csv file.

## 4 Online LD Analysis

### 4.1 Proof of Principle Using Particle Size Standards

**Figure 8:**
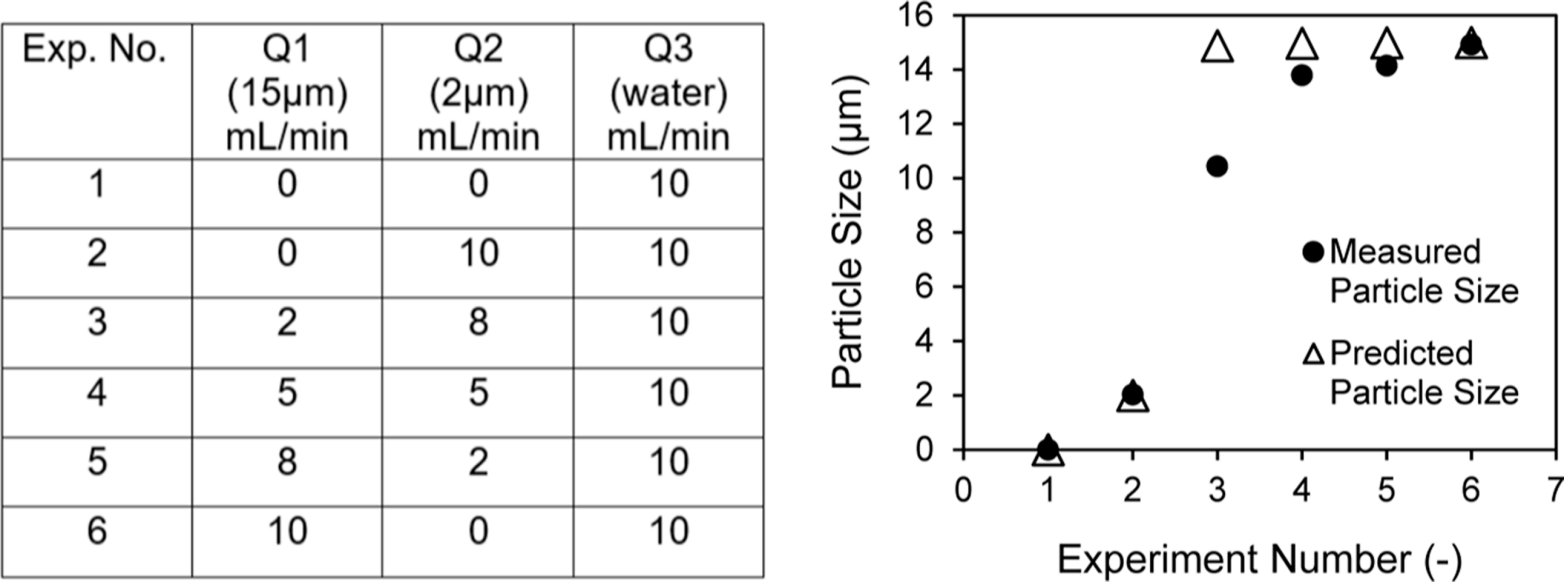
Comparison of measured mean particle sizes obtained via online LD and predicted mean particle sizes for particle standards mixed in different ratios (Q1: 15 μm suspension flow rate, Q2: 2 μm suspension flow rate, and Q3: water flow rate).

For validating the online LD analysis technique, suspensions of standard polystyrene microparticles (15 and 2 μm) in DI water were mixed using a Y-junction and flown to the LD flow cell via peristaltic pumps. The flow rate of standard particle suspensions was varied to form suspensions having different ratios of the 15 and 2 μm particle standards (Figure 8). The measured particle sizes of suspensions of different particle size ratios using the online LD platform are presented in Figure 8 and compared with predicted mean particle sizes. The De Brouckere mean diameter,^37^*D*(4,3), which is defined as the ratio between the fourth and third moments of the PSD, was used. The predicted mean particle sizes are in agreement with the majority of the experiments. But for bimodal PSDs, the measured mean particle sizes are consistently lower than the predicted values.

#### 4.1.1 Characterization of the Ibuprofen Suspension Produced via Antisolvent Crystallization in the CIJR

The automated crystallization platform was employed for antisolvent crystallization of ibuprofen. A concentration of 39.45 mg/mL ibuprofen was chosen for the flow experiments, as it was found to be the highest concentration that could be used without resulting in particle aggregation and clogging of the crystallization platform. The antisolvent flow rate was 4 mL/min, while the antisolvent/solvent ratio (that is, the ratio of antisolvent flow rate to solvent solution flow rate) was varied between 5 and 9. These conditions were chosen to avoid aggregation and fouling in the CIJR.

**Figure 9:**
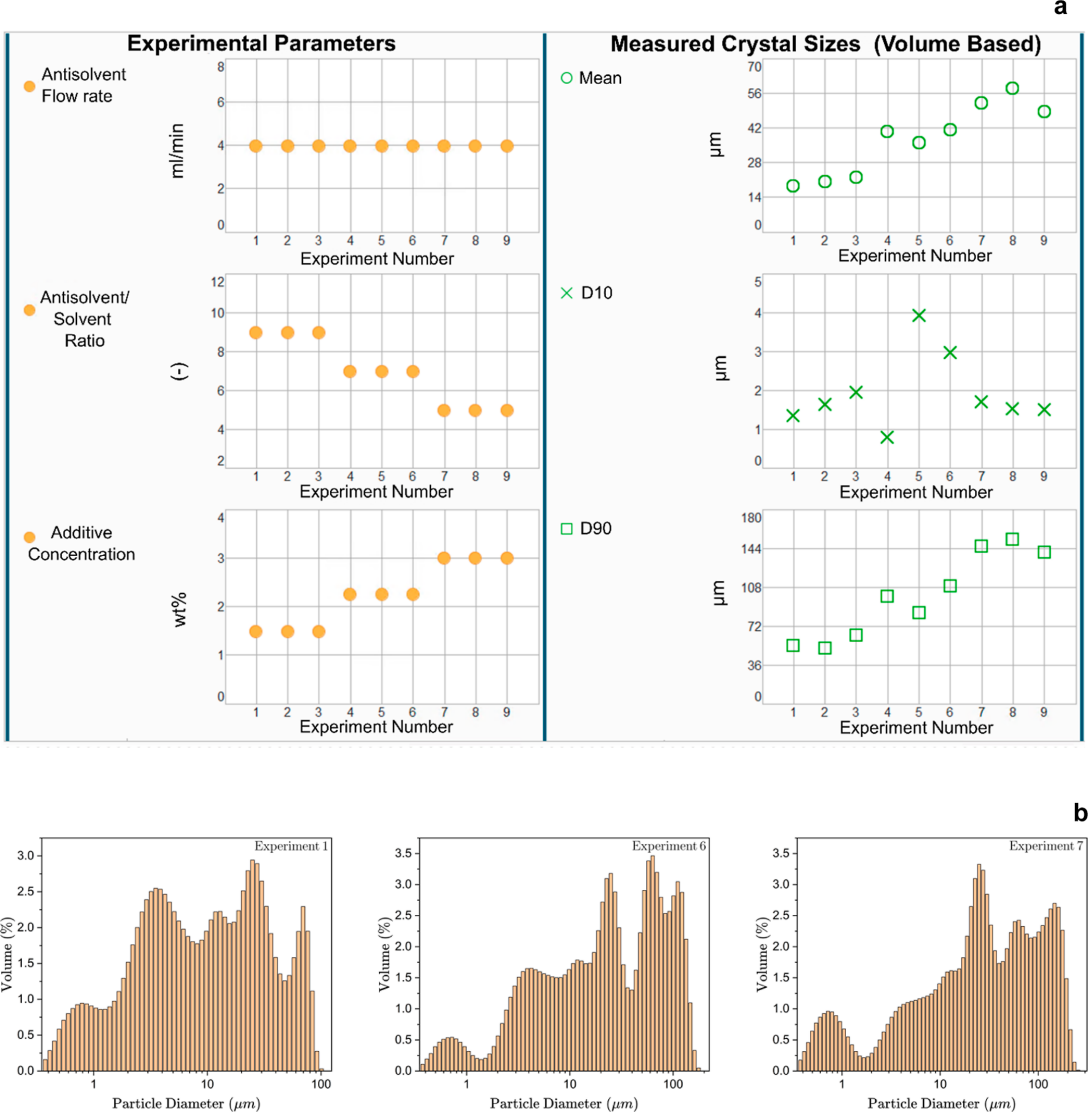
(a) Crystal size (mean, D10, and D90) of ibuprofen suspensions at different experimental conditions in the PharmaMV GUI using the volume-based crystal size distribution. (b) Particle size distribution of ibuprofen suspensions at different experimental conditions (for experiments nos 1, 6, and 7) using volume-based crystal size distributions.

The Soluplus additive was used for growth prevention and stabilization of the crystals. Figure 9a presents the GUI developed in PharmaMV, showing the experimental parameters of interest (i.e., antisolvent flow rate, antisolvent/solvent ratio, and additive concentration) and the resulting crystal size (mean, D10, and D90) using the volume-based crystal size distributions. The front panel was designed to update automatically to reflect any changes in the crystal size in the suspension. Experiments were performed under three different sets of conditions with three replicates. Figure 9b shows the particle size distributions of ibuprofen under the three experimental conditions represented in Figure 9a.

The standard deviation of the particle sizes varied from 1.6 μm (7.81%) for experiments 1–3 to 2.74 μm (6.93%) for experiments 4–6 and to 4.77 μm (8.97%) for experiments 7–9 (see Figure 9a for the experimental conditions). The standard deviation of the measured particle sizes using the automated online LD platform was less than 10%, showing satisfactory reproducibility of the measurements.

The volume-based PSD (Figure 9b) is broad, with multiple peaks. This suggests the polydispersity of the crystal sizes, confirmed by optical microscopy images (see Figure S3 in the Supporting Information). Many small particles in the submicron range are present, along with larger particles with size below 10 μm. However, a few larger crystals and a certain degree of agglomeration can be clearly observed in the images. Note that volume-based size distributions are biased toward larger sizes, a bias that is considerable even if few large crystals are present.^26^ Despite generation of ibuprofen crystals in the submicrometer range, the presence of larger crystals and crystal agglomerates drives the mean crystal sizes obtained from LD. The larger particles and agglomerates do not influence as much the number-based crystal size distributions obtained from the same experiments (see Figure S2), which only reveal the presence of particles smaller than 10 μm. We can conclude that the volume-based distribution overestimated the particle sizes due to the presence of agglomerates.

Increasing the antisolvent/solvent ratio was found to reduce the mean particle size, a finding that could be due to higher supersaturation ratios, resulting in faster nucleation rates. Increasing the additive concentration beyond 1.5 wt % was not found to be effective in stabilizing the ibuprofen crystals more. Further investigation, based on the DoEs, the identification of fouling-free feasible operating areas, and the effect of operating parameters on the crystal size of ibuprofen, is ongoing but exceeds the scope of this work. Based on the automated crystallization platform described, the development of a closed-loop antisolvent crystallization platform with the capability to self-optimize the process parameters for desired particle properties is also currently under progress.

The LD system used in our work has a lower limit of a 400 nm particle size. However, state-of-the-art LD analyzers with PIDS technology can measure particle sizes up to 10 nm. Dynamic light scattering, which is the more suitable and commonly used technique for measuring particle size in the nanoparticle range, also requires online sample dilution to be implemented in an online fashion.

## 5 Conclusions

The automated continuous crystallization platform developed using LabVIEW, Python, and PharmaMV can perform sets of predetermined, user-controlled experiments while measuring and displaying PSDs in real time without any user involvement. The automated online dilution system of the crystal suspension obtained from the crystallizer enabled reproducible LD measurements without multiple scattering. The platform was showcased to obtain 20–50 μm-sized ibuprofen crystals using a CIJR. The modular plug-and-play nature of the platform can allow using different types of crystallizers, such as CSTRs, and other laser-based crystal characterization technologies, such as dynamic light scattering and Raman spectroscopy. The fully automated nature of the crystallization platform, along with the online implementation of the LD analyzer as a PAT, can enable rapid screening of process parameters and identify conditions suitable for obtaining the desired size of API crystals, thereby expediting the process development for antisolvent crystallization in industrial applications.

## Supporting Information Available

VI development; data communication protocol and SVE; number-based crystal size distribution of ibuprofen suspensions; optical microscopy of ibuprofen suspensions; and process flow diagram for production (PDF)
