# Supplementary material for: Automated Continuous Crystallization Platform with Real-Time Particle Size Analysis via Laser Diffraction
Source: Org Process Res Dev. 2024 Jul 9;28(7):2755–64. doi: 10.1021/acs.oprd.4c00110 (PMC11267596; doi:10.1021/acs.oprd.4c00110)
Supplement: Supplementary file 1 — op4c00110_si_001.pdf [file op4c00110_si_001.pdf]

Supplementary Information

Automated continuous crystallization platform with real-time particle size analysis via laser diffraction

Sayan Pal [a], Arun Pankajakshan [a], Maximilian O. Besenhard [a],
Nicholas Snead [a], Juan Almeida [b], Shorooq Abukhamees [c], Duncan Craig [d],
Federico Galvanin [a], Asterios Gavriilidis [a], Luca Mazzei [a]

[a] Department of Chemical Engineering, University College London, Torrington Place, London, WC1E 7JE, United Kingdom

[b] Perceptive Engineering, Applied Materials, Vanguard House, Keckwick Lane, Sci Tech Daresbury, Cheshire WA4 4AB, United Kingdom

[c] Department of Pharmaceutics and Pharmaceutical Technology, Faculty of Pharmaceutical Sciences, The Hashemite University, Zarqa 13115, Jordan

[d] Faculty of Science, University of Bath, Claverton Down, Bath, BA2 7AY, United Kingdom

S1. Virtual Instrument (VI) Development

In the code snippet shown in Figure 4 (of the main manuscript), LabVIEW functions were employed to streamline the process. The 'Build Path' function generates a path to the specified instrument VI using a string input (VI's name). The 'Open VI Reference' function then retrieves a reference to that particular instrument VI. Subsequently, the 'Invoke Node' function is used with the 'FP.Open' method to open the front panel window of the instrument VI and set its state as specified by the connected 'State' input (usually minimized by default). Finally, the 'Invoke Node' function with the 'Run VI' method executes the VI. The advantage of this approach lies in the capability of the master VI to control all the instrument VIs. By simply opening and running the master VI, all connected instrument VIs are executed automatically. The front panel of the master VI contains controls and indicators for the instrument VIs (Figure 4), along with two types of stop buttons: one to halt the execution of any specific instrument VI and the other, called 'TERMINATOR', to stop all VIs simultaneously. This architecture allows for a more elegant and efficient way to automate instrument functions using LabVIEW. The front panel of the master VI developed to control the equipment in the crystallization platform is shown in Figure S1.

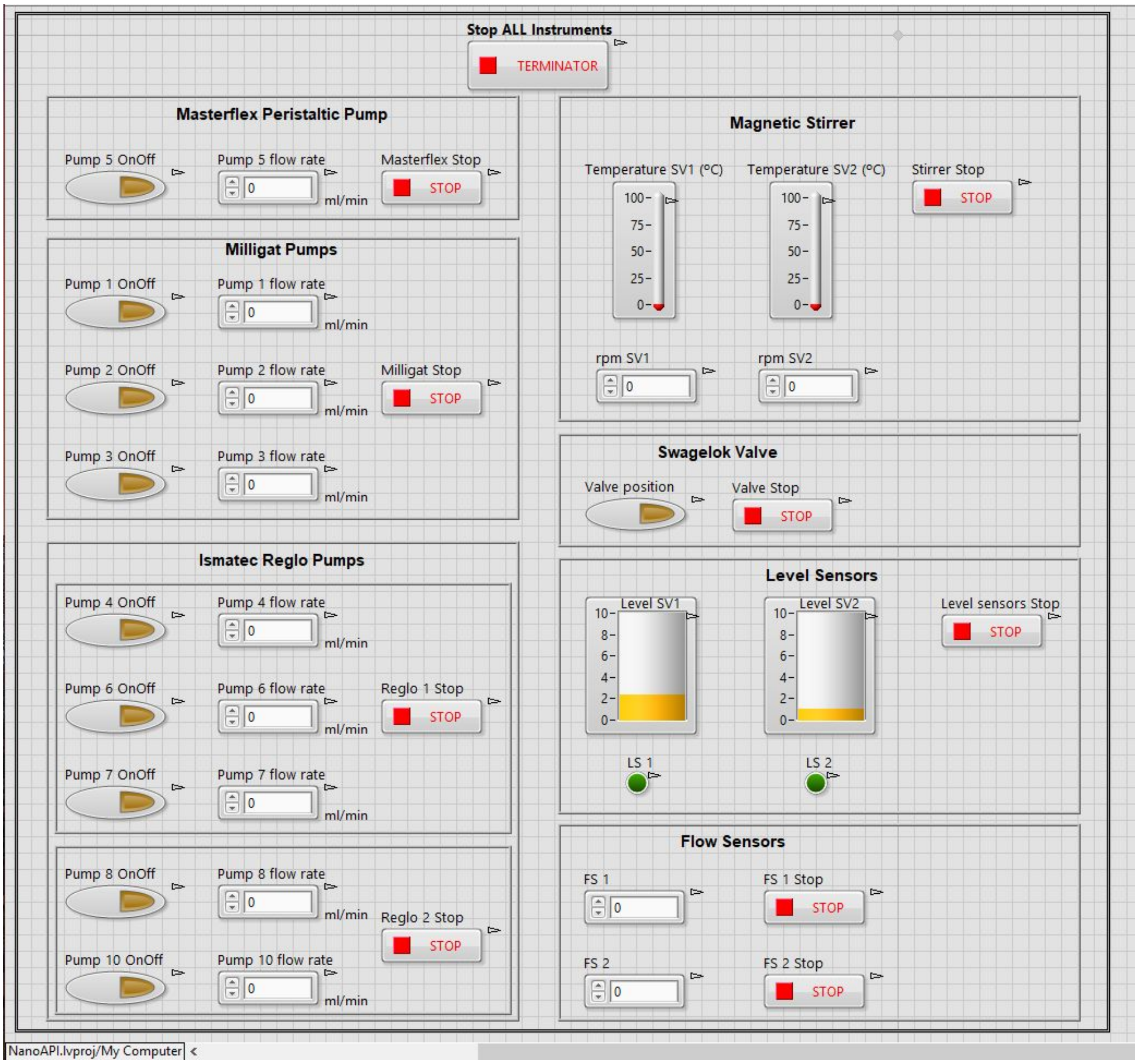

Figure S1. Front panel of the LabVIEW master VI.

S2. Data Communication Protocol and Shared Variable Engine (SVE)

The Shared Variable Engine is a software component installed as a service along with LabVIEW installation and uses National Instruments Publish-Subscribe-Protocol (NI-PSP)[1] to manage shared variable updates. Before shared variables are read or written over the network, they must be deployed to the SVE, which creates a memory space for them. When a VI that uses shared variables is executed, LabVIEW automatically deploys the project library containing the shared variables to the SVE and the SVE starts working as a separate process on the computer. This step is illustrated in Figure 6 (of the main manuscript). The SVE also acts as an OPC server [2], which is a standard interface to communicate between various data sources such as factory equipment, laboratory devices, databases etc.

S3. Number-Based Crystal Size Distribution of Ibuprofen Suspensions

Figure S2 shows the comparison of the number-based crystal size distributions for the three experimental conditions considered in Figure 9 (experiments 1, 6 and 7). The number-based distribution was obtained by converting the volume-based distribution originally measured from the laser diffraction analyser. The larger particles and the agglomerates do not influence as much the number-based crystal size distributions (see Figure S2), which only feature particles smaller than 10 μm.

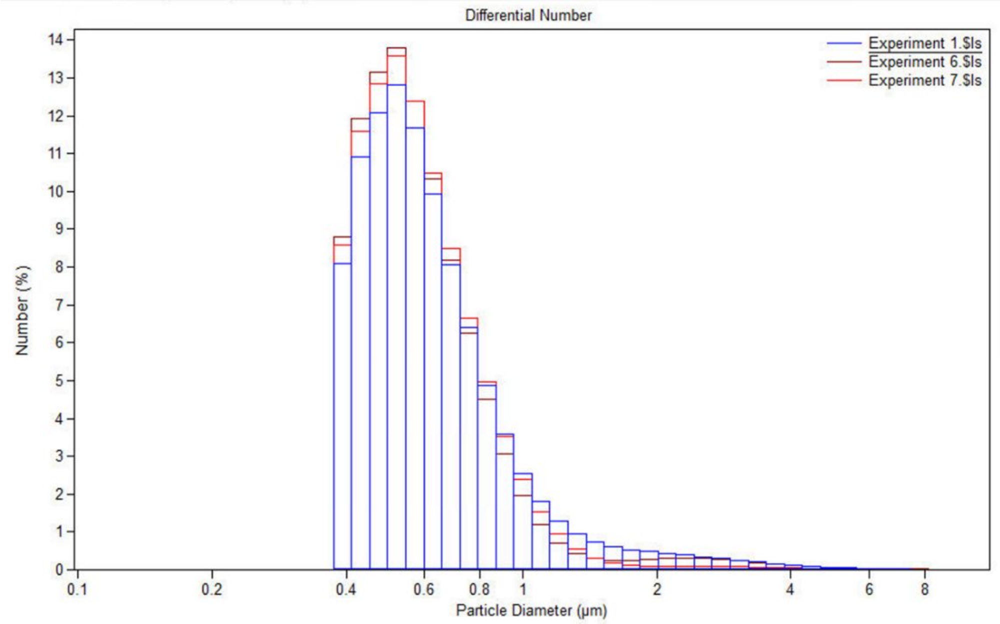

Figure S2. Number-based crystal size distributions of ibuprofen suspensions at different experimental conditions.

S4. Optical Microscopy of Ibuprofen Suspensions

Figure S3 shows the optical micrographs of an ibuprofen suspension taken employing a Keyence VHX 7000 microscope. The micrograph confirms that the ibuprofen crystal suspension contains a small population of large crystals and a large population of small crystals.

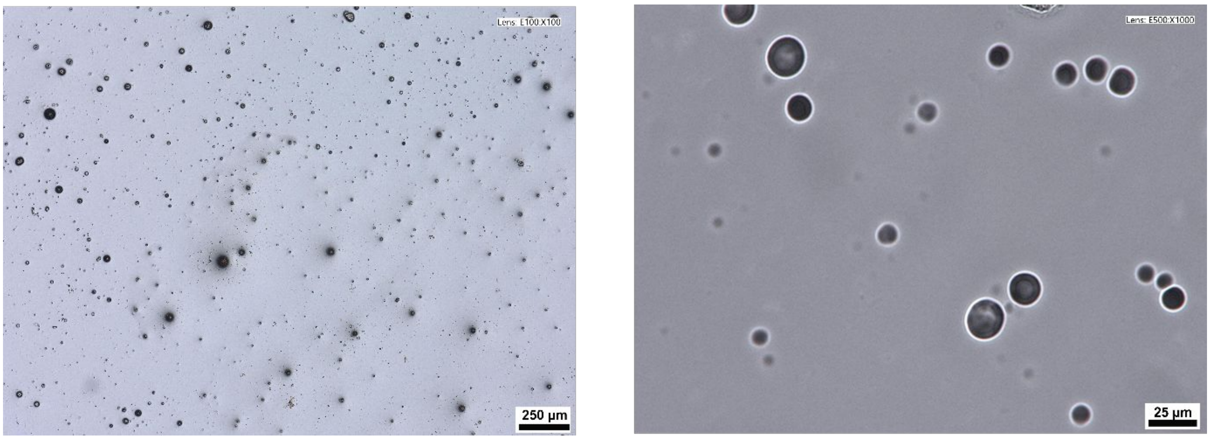

Figure S3. Optical microscopy images of the ibuprofen suspension obtained from experiment 1 (antisolvent flow rate 4 ml/min; antisolvent/solvent ratio 9; additive concentration 1.5 wt%).

S5. Process Flow Diagram for Production

After the screening of the operating parameters was completed, the continuous crystallization platform was operated entirely for production without involving the analysis section of the process flow diagram. A separate collection bottle was added at the end of blue line, keeping valve V1 in that direction. The setup can be very easily modified based on the goal - screening or production.

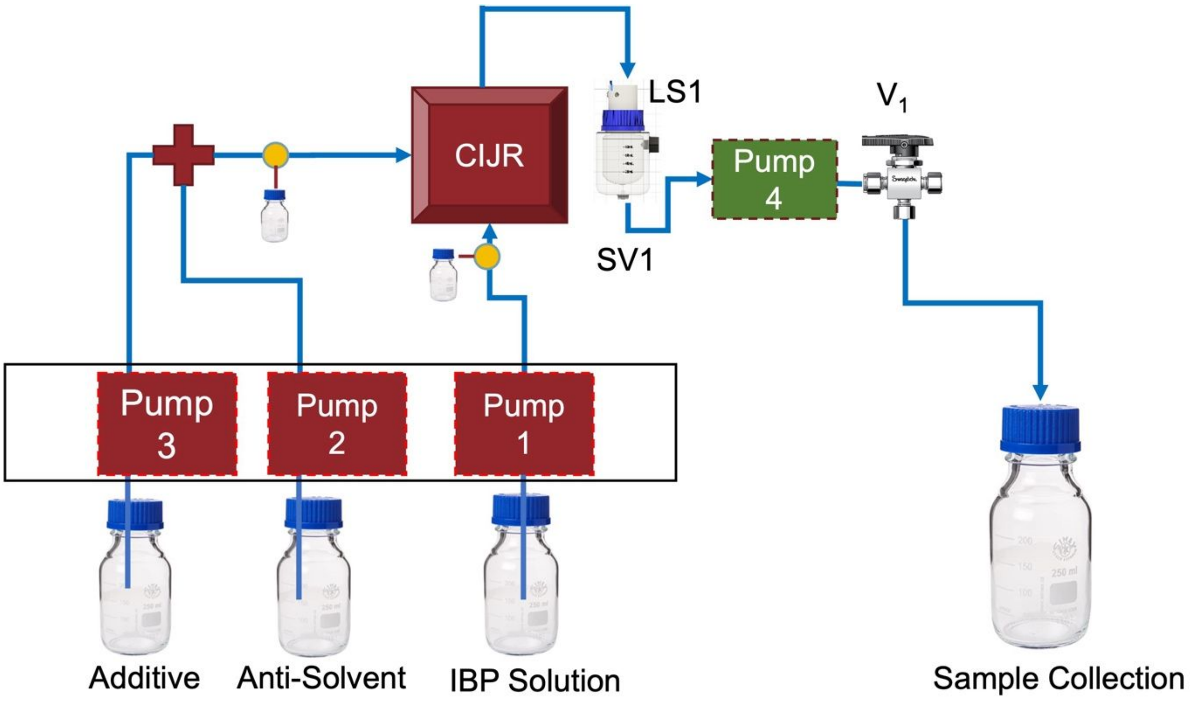

Figure S4. Process flow diagram of the crystallization platform for production without online laser diffraction.

References

(1) *Buffered Network-Published Shared Variables: Components and Architecture - NI*. https://www.ni.com/en/support/documentation/supplemental/22/buffered-network-published-shared-variables--components-and-arch.html (accessed 2023-11-28).

(2) *What is OPC? - OPC Foundation*. https://opcfoundation.org/about/what-is-opc/ (accessed 2023-07-23).
